# Supplementary figures and images for: c-Jun N-terminal kinase (JNK) signaling contributes to cystic burden in polycystic kidney disease
Source: PLoS Genet. 2021 Dec 28;17(12):e1009711. doi: 10.1371/journal.pgen.1009711 (PMC8746764; doi:10.1371/journal.pgen.1009711)

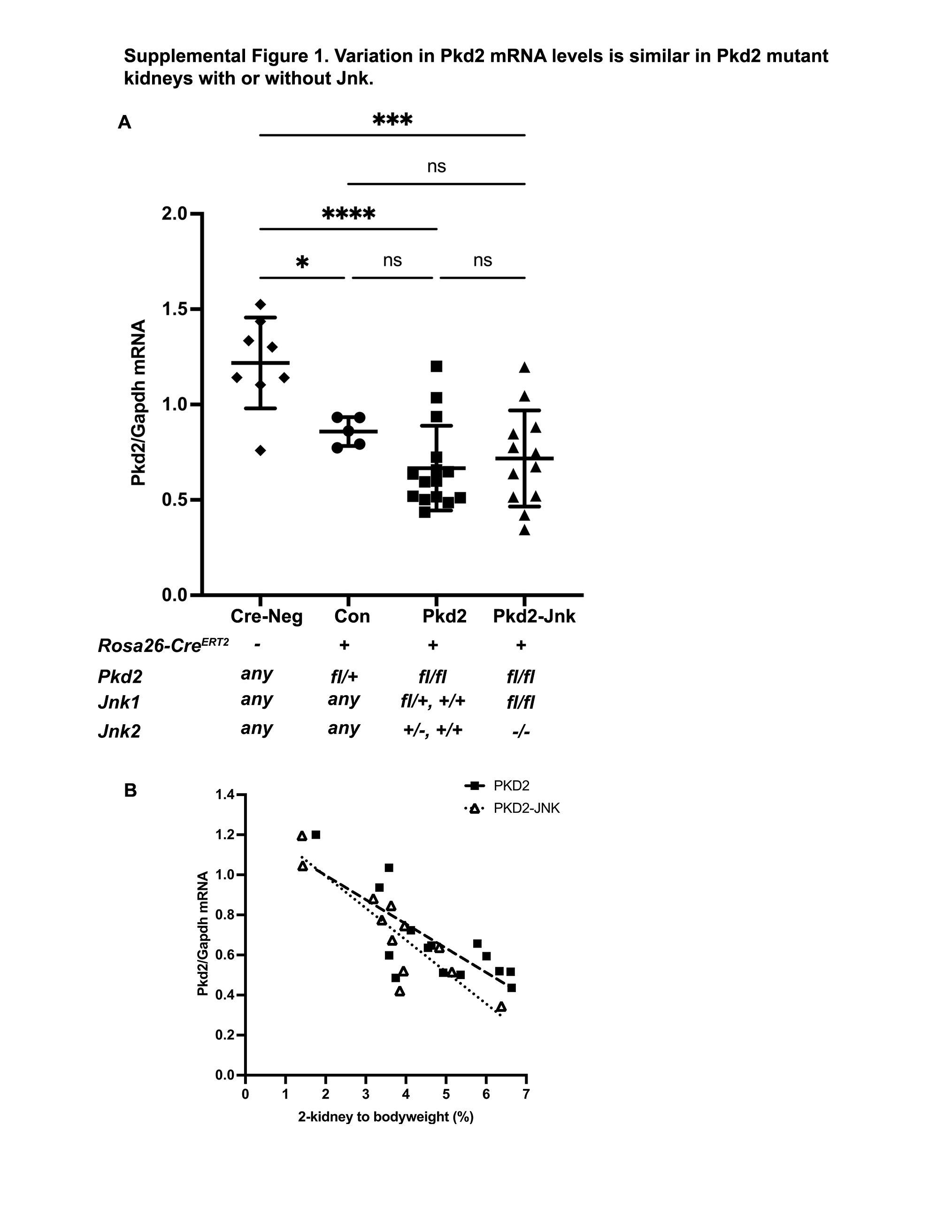

Supplement: S1 Fig — (A) Total RNA was extracted from P21 kidneys. Pkd2 mRNA levels were determined by RT-qPCR and normalized to Gapdh. All animals were treated with tamoxifen by maternal oral gavage on P2-4. The groups consist of Cre-negative animals (N = 8), Pkd2 heterozygotes with any number of Jnk alleles (Rosa26-CreERT2; Pkd2fl/+) (N = 5), Pkd2 mutants with at least one wild type allele of Jnk1 and Jnk2 (Rosa26-CreERT2; Pkd2fl/fl) (N = 15), and Pkd2 mutants with no functional Jnk alleles (Rosa26-CreERT2; Pkd2fl/fl; Jnk1fl/fl; Jnk2null/null) (N = 12). ****, P < 0.0001; ***, P < 0.001; *, <0.05; ns, not significant by one-way ANOVA followed by Tukey multiple comparison test with multiplicity-adjusted p-values. Error bars indicate SD. (B) Data from A replotted against 2-kidney to body weight. Pkd2 mRNA levels were plotted against 2-kidney to body weight for individual Pkd2 mutants with at least one wild type allele of Jnk1 and Jnk2 (PKD2, N = 15) or lacking all functional Jnk alleles (PKD2-JNK, N = 12). Each group was analyzed by simple linear regression. For a given reduction in Pkd2 mRNA, mutants lacking Jnk tend to have a lower cystic burden. However, the slopes of the two lines were not statistically significantly different. PKD2: Y = -0.1212*X + 1.240, PKD2-JNK: Y = -0.1593*X + 1.312. (TIF) [file pgen.1009711.s001.tif]

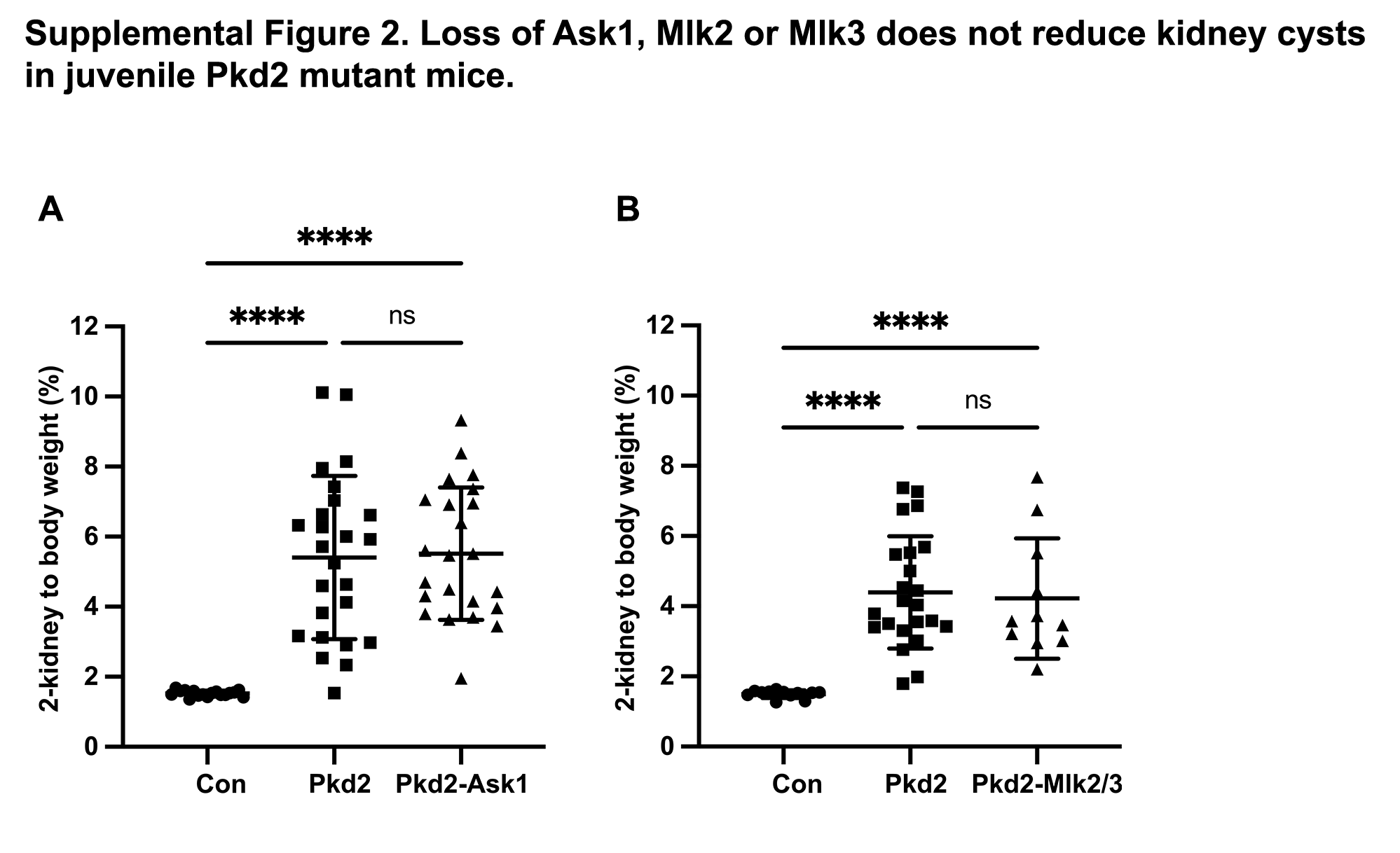

Supplement: S2 Fig — Mice with the following genotypes were treated with tamoxifen at P2-4 and collected at P21: Con (Rosa26-CreERT2; Pkd2fl/+), Pkd2 (Rosa26-CreERT2; Pkd2fl/fl), Pkd2-Ask1 (Rosa26-CreERT2; Pkd2fl/fl; Ask1-/-) and Pkd2-Mlk2/3 (Rosa26-CreERT2; Pkd2fl/fl; Mlk2-/-; Mlk3-/-). (A) Cystic burden was quantified using the ratio of 2-kidney weight / body weight x 100%. N is 17 (Con), 25 (Pkd2), 23 (Pkd2-Ask1). ****, P < 0.0001; ns, not significant by one-way ANOVA followed by Tukey multiple comparison test with multiplicity-adjusted p-values. Error bars indicate SD. (B) Cystic burden was quantified using the ratio of 2-kidney weight / bodyweight x 100%. N is 12 (Con), 23 (Pkd2), 11 (Pkd2-Mlk2/3). ****, P < 0.0001; ***, P < 0.001; ns, not significant by one-way ANOVA followed by Tukey multiple comparison test with multiplicity-adjusted p-values. Error bars indicate SD. (TIF) [file pgen.1009711.s002.tif]

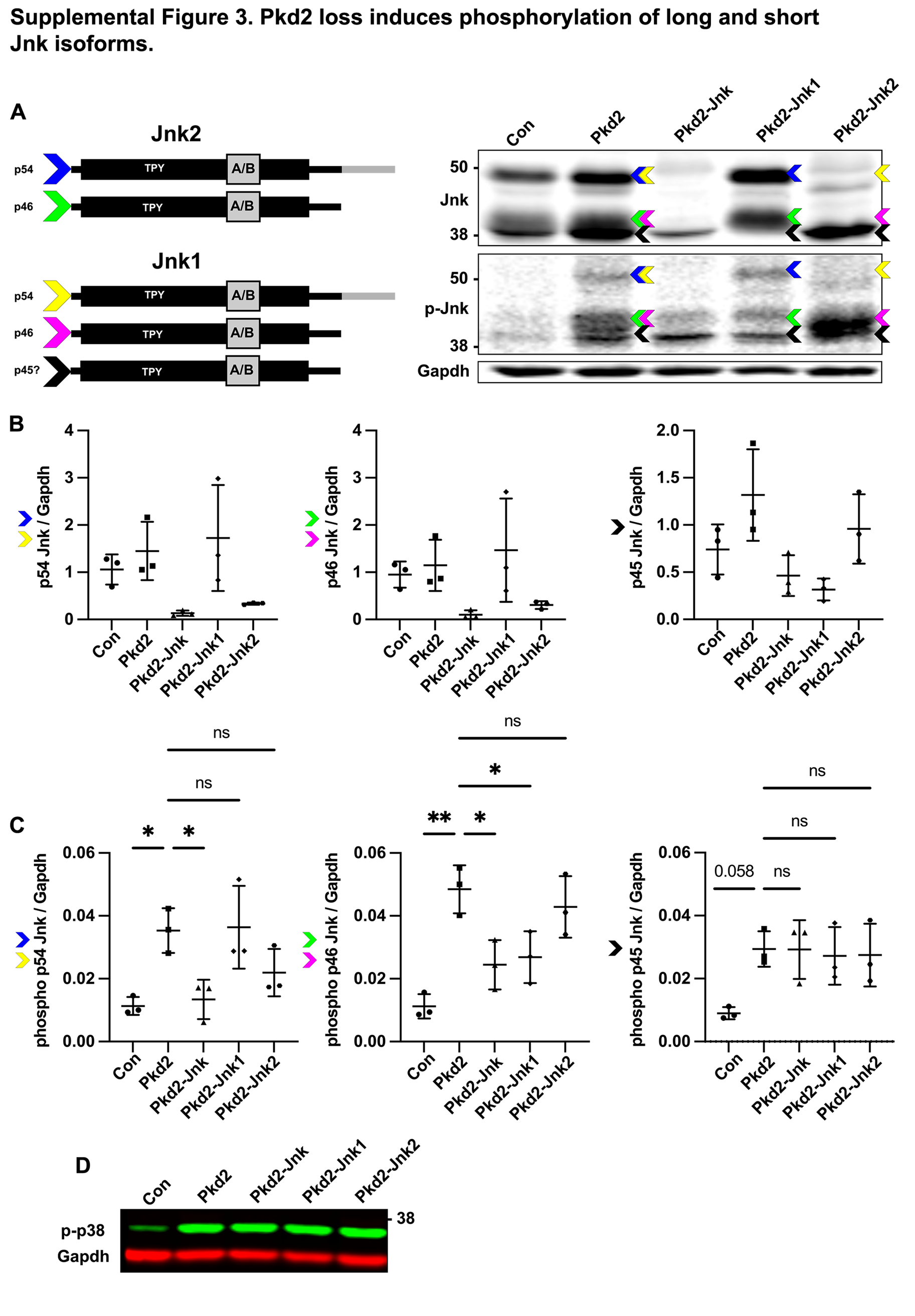

Supplement: S3 Fig — Mice described in Fig 2: Con (Rosa26-CreERT2; Pkd2fl/+), Pkd2 (Rosa26-CreERT2; Pkd2fl/fl; JNK1+/+; Jnk2+/+), Pkd2-Jnk (Rosa26-CreERT2; Pkd2fl/fl; Jnk1fl/fl; Jnk2null/null) Mice described in Fig 6: Pkd2-Jnk1 (Rosa26-CreERT2; Pkd2fl/fl; Jnk1null/null; Jnk2+/+); Pkd2-Jnk2 (Rosa26-CreERT2; Pkd2fl/fl; JNK1+/+; Jnk2null/null). (A) Whole kidney protein lysates were immunoblotted for total Jnk, phospho T183/Y185 Jnk, and loading control Gapdh. The diagram (left) depicts the primary known isoforms of Jnk1 and Jnk2, as well as a putative isoform of Jnk1. Both Jnk1 and Jnk2 can be alternatively spliced to form four variants. Incorporation of the mutually exclusive exon pair 7a/7b in the kinase domain does not affect size of the protein, but alternative splicing at the C-terminus can produce either long (p54) or short (p46) forms that can be distinguished by immunoblot. Colored arrows indicate corresponding isoforms on the blot. These represent our predictions of which isoforms are present in each band. The black arrows correspond to a band of unknown identity (p45) which we quantified separately. (B-C) Quantification of immunoblots described in (A). N is 3 animals per group. Due to low sample number, differences in total Jnk isoform levels did not reach statistical significance in most cases and are not displayed. For phosphorylated Jnk isoforms, we indicate the significance of each group compared to Pkd2 mutants. Phospho-p45 nearly reached significance with p = 0.058. **, P < 0.01; *, P < 0.05; ns, not significant by one-way ANOVA followed by Tukey multiple comparison test with multiplicity-adjusted p-values. Error bars indicate SD. (D) The kidney lysates described in (A) were immunoblotted for phospho T180/Y182 p38 and loading control Gapdh. (TIF) [file pgen.1009711.s003.tif]

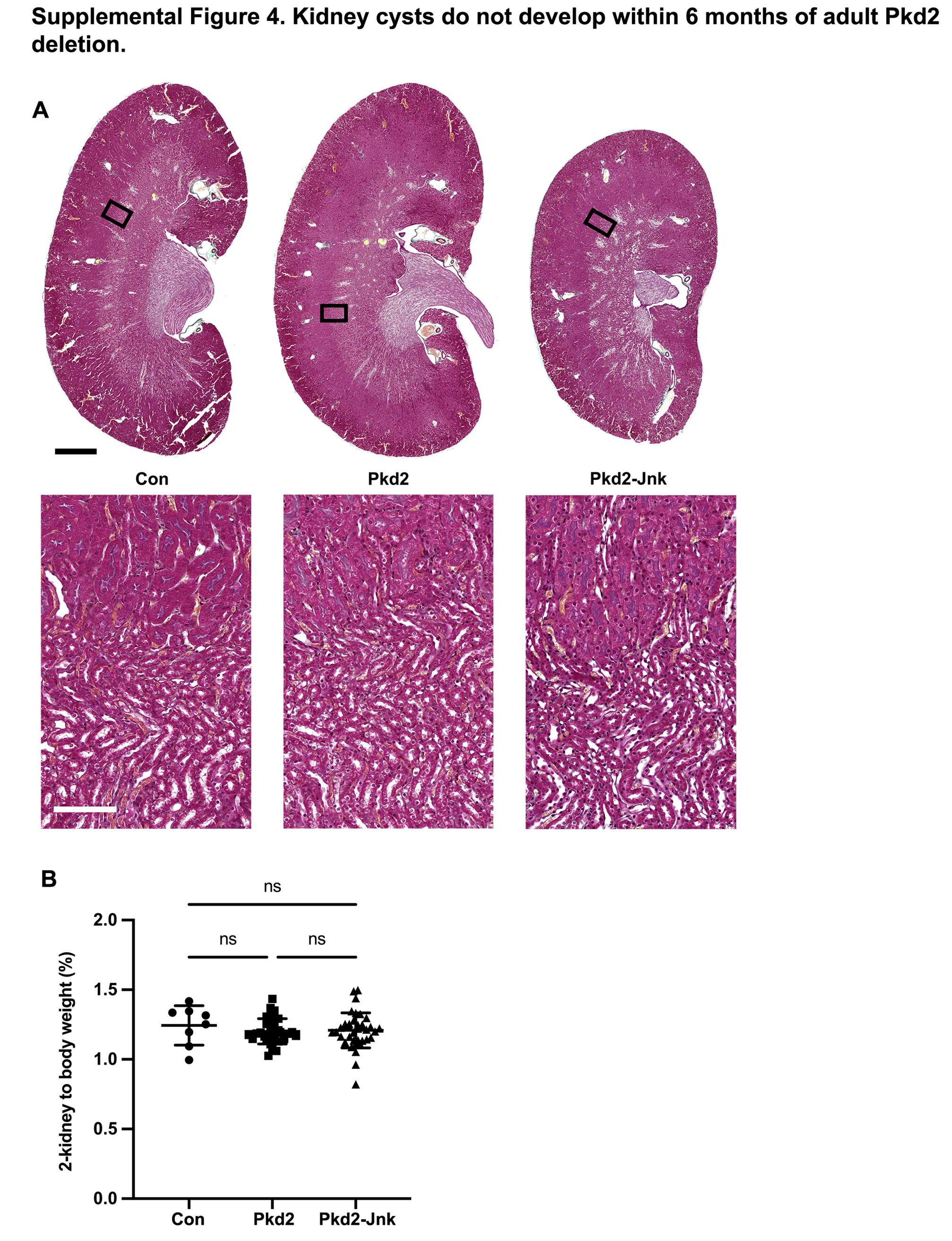

Supplement: S4 Fig — Mice with the following genotypes were treated with tamoxifen at P21-23 and collected 24 weeks later: Con (Rosa26-CreERT2; Pkd2fl/+), Pkd2 (Rosa26-CreERT2; Pkd2fl/fl; Jnk1+/+, fl/+; Jnk2+/+, +/-), Pkd2-Jnk (Rosa26-CreERT2; Pkd2fl/fl; Jnk1fl/fl; Jnk2null/null). (A) Kidney sections were stained with one-step trichrome to mark collagen fibers pale green, cytoplasm red, and nuclei dark blue. Scale bar is 1000 microns for full size kidney scans, 100 microns for insets. (B) Cystic burden in the kidney was quantified using the ratio of 2-kidney / body weight x 100%. N is 8 (Con), 31 (Pkd2), 38 (Pkd2-Jnk). ns, not significant by one-way ANOVA followed by Tukey multiple comparison test with multiplicity-adjusted p-values. Error bars indicate SD. (TIF) [file pgen.1009711.s004.tif]

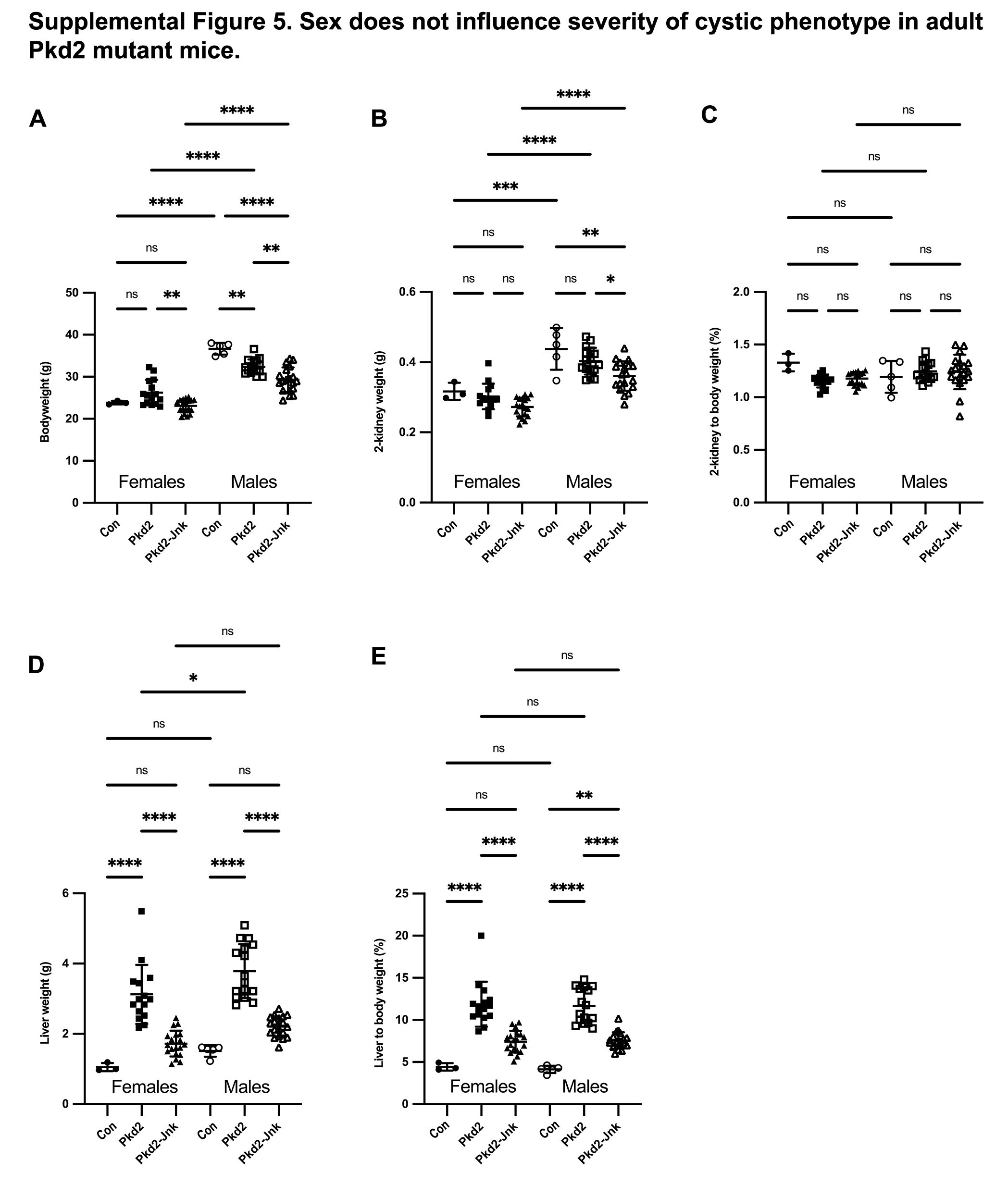

Supplement: S5 Fig — Mice with the following genotypes were treated with tamoxifen at P21-23 and collected 24 weeks later: Con (Rosa26-CreERT2; Pkd2fl/+), Pkd2 (Rosa26-CreERT2; Pkd2fl/fl; Jnk1+/+, fl/+; Jnk2+/+, +/-), Pkd2-Jnk (Rosa26-CreERT2; Pkd2fl/fl; Jnk1fl/fl; Jnk2null/null). (A) Body weight of adult Pkd2 mutant mice, segregated by sex. Females: N is 3 (Con), 15 (Pkd2), 19 (PKD2-JNK). Males: N is 5 (Con), 16 (Pkd2), 19 (Pkd2-Jnk). ****, P < 0.0001; **, P < 0.01; ns, not significant by one-way ANOVA followed by Tukey multiple comparison test with multiplicity-adjusted p-values. Error bars indicate SD. (B-C) 2-kidney weight and 2-kidney to body weight (%) of adult Pkd2 mutant mice, segregated by sex. Females: N is 3 (Con), 15 (Pkd2), 19 (PKD2-JNK). Males: N is 5 (Con), 16 (Pkd2), 19 (Pkd2-Jnk). ****, P < 0.0001; ***, P < 0.001; **, P < 0.01; *, P < 0.05; ns, not significant by one-way ANOVA followed by Tukey multiple comparison test with multiplicity-adjusted p-values. Error bars indicate SD. (D-E) Liver weight and liver to body weight (%) of adult Pkd2 mutant mice, segregated by sex. Females: Females: N is 3 (Con), 15 (Pkd2), 19 (PKD2-JNK). Males: N is 5 (Con), 16 (Pkd2), 19 (Pkd2-Jnk). ****, P < 0.0001; **, P < 0.01; ns, not significant by one-way ANOVA followed by Tukey multiple comparison test with multiplicity-adjusted p-values. Error bars indicate SD. (TIF) [file pgen.1009711.s005.tif]
