# Supplementary material for: c-Jun N-terminal kinase (JNK) signaling contributes to cystic burden in polycystic kidney disease
Source: PLoS Genet. 2021 Dec 28;17(12):e1009711. doi: 10.1371/journal.pgen.1009711 (PMC8746764; doi:10.1371/journal.pgen.1009711)
Supplement: Attachment — Submitted filename: ResponseToReviewers_12_05_2021.pdf [file pgen.1009711.s007.pdf]

Reviewer #1: All questions have been answered.

Reviewer #2: Have address key concerns of this reviewer.
Look forward to follow-up mechanistic studies and therapeutic implications.

Reviewer #3: The authors addressed most of my concerns. I apologize that I did not understand the "making-of" the Jnk1 null/null line correctly. Of course, the authors are right, no problem in using this - based on the very same allele.

I am still unhappy with the label "Wt" in several figures refering to heterozygous knockout animals.

“Wt” was changed to “Con” at all instances in the manuscript, figure legends and figures.

In addition, Fig. S6 and S7 should be either rearranged or deleted.

Fig. S6 and S7 were removed.
